# Analysis of Influencing Factors of College Students’ Intention to Repeated Blood Donation Based on Structural Equation Modeling

**DOI:** 10.1007/s12288-021-01455-4

**Published:** 2021-06-26

**Authors:** Lingling Pan, Wei Hu, Wenjuan Han, Yingying Wang

**Affiliations:** 1 Blood Center of Zhejiang Province, Hangzhou, China; 2 Key Laboratory of Blood Safety Research, Zhejiang Province, Hangzhou, China

**Keywords:** College students, Repeated blood donation, The theory of planned behavior, Structural equation modeling

## Abstract

To research the influencing factors of college students' blood donation behavior intention and propose intervention strategies to improve the repeated blood donation rate of college students. Questionnaire survey was used to research and analyze the influencing factors of behavior intention. Amos 21.0 software was used to establish structural equation modeling and perform confirmatory factor analysis. SPSS 20.0 was used for statistic. The model was proved with highly adaptability, with χ^2^/df = 2.956 < 3. Factors influencing college students' intention of repeat blood donation behavior can be summarized into four: attitude, external motivation, advice-taking, and perceived behavioral control. Among them, attitude and perceived behavioral control have a great direct impact on behavioral intention, while the external motivation and recommendation acceptance have an indirect impact by influencing the other two factors. In view of those evaluation items with high path coefficient in each factor, we can develop recruitment strategies to influence college students’ repeated blood donation behavior and provide scientific suggestions for improving their repeated blood donation rate.

## Introduction

In recent years, the amount of blood transfusions and voluntary non-remunerated blood donors have been raising continuously in China. In 2018, China made more than 15 million voluntary non-remunerated donations, with the amount of blood donated reaching 5 billion milliliters. All clinical blood transfusion in China comes from voluntary non-remunerated blood donations now. As elsewhere in the world, college students are an important force to be reckoned with in the blood donors [1]. In our previous studies, the proportion of college students in blood donors fluctuated frequently and the repeated blood donation rate was low in Hangzhou, Zhejiang province. In 2009, 15.05% of blood donors in Hangzhou were college students. In 2012, this proportion dropped to 9.36%, and gradually increased from 2013. In terms of repeated blood donation rate, this ratio of college students in Hangzhou was only 5.5% in 2019, which was far lower than that in Zhejiang province (30.8%). Therefore, studies on improving blood donation rate of college students, especially the repeated blood donation rate, can help to promote blood donation in Hangzhou.

According to the theory of planned behavior, information processing, analysis and thinking will be carried out before behavior occurs, all factors interact to ultimately determine the motivation of people's behavior [2–5]. Behavioral intention, a variable that directly determines individual behavior, is affected by attitude, subjective norms and perceived behavioral control on the other side [6–9]. To repeated blood donation behavior, there are a number of influencing factors. In the past, most of studies have focused on analyzing the direct effects of influencing factors on repeated donation behavior, while fewer talked about indirect effects and interactions between factors.

Therefore, we carried out this study based on the theory of planned behavior, to comprehensively evaluate the effects of influencing factors on repeated blood donation behavior by conducting questionnaire survey and establishing structural equation modeling. As a result, we want to better understand the internal psychological mechanisms that influencing the behavioral intention of college students, so as to provide scientific suggestions for improving the repeated blood donation rate among them [10].

## Participants and Methods of the Study

### Questionnaire

The questionnaire was designed based on a previous study we conducted: Establishment of the index system for the investigation and evaluation of repeated blood donation behavior intention and related influencing factors. Experts were invited to evaluate the importance of 35 items in the index system, and items with an average score above 6 were retained. Correlation analysis was performed on the retention items to remove those whose correlation coefficients were lower than 0.5. Conducted reliability analysis on the remaining items and proved have a high reliability of internal consistency, with the Guttman half-fold coefficient was 0.940 and Cronbach’s α was 0.973. Finally, 31 items were determined to make up the questionnaire, including 8 attitude items, 10 subjective behavior norms items, 11 perceived behavior control items, and 2 behavioral intention items [6].

### Study Participants

In 2018, a total of 38 schools in Hangzhou carried out blood donation drives in students, including 20 universities and 18 higher vocational colleges. We selected 8 schools by random sampling from them. Statistical analysis showed that there was no statistically significant difference between the randomly selected schools and the population in terms of school types (χ2 = 1.346, *p* = 0.435).

A random online questionnaire survey was conducted on college students who donated blood in these 8 schools from November 21, 2018 to December 20, 2018. A total of 842 questionnaires were submitted, 670 (79.57%) of which were filled in completely and deemed valid.

### Statistical methods

SPSS 20.0 was used to perform chi-square test, correlation analysis, principal component analysis, exploratory factor analysis, etc. Confirmatory factor analysis and model establishment were performed by using Amos 21.0 software.

In this study, we applied a multivariate statistical method called structural equation modeling. It can use linear equations to explain the relationship between observed variables and latent variables, as well as the relationship between latent variables. We put a number of variables affecting the repeated blood donation behavior of college students into model simultaneously and observed their interaction and the direct and indirect effects on results. Then, exploratory factor analysis and model adaptability test were used to verify the adaptability of the model. Maximum Likelihood was used to calculate path coefficient between variables, which can quantitatively evaluate the effect of variables in the model.

## Results

### Study Participants

A total of 670 valid participants, with the age of 20.05 ± 1.18 (mean ± SD) years, of which 580 (86.57%) were first-time donors and 90 (13.43%) donated blood at least twice. Of those, 298 (44.5%) were male and 372 (55.5%) were female; 454 (67.8%) from universities and 216 (32.2%) from higher vocational colleges. (Tables 1,2) Doing statistical analysis between study participants and total population, and it was found that there was no statistically significant difference between them in terms of gender and blood donation times. (Table 3) This indicates that the students we randomly selected represent the overall situation of college blood donation in Hangzhou during 2018.

**Table 1:** Distribution of blood donation times

Donation frequency	Number of blood donors (*n*, %)
First-time	580 (86.57)
Repeated	90 (13.43)
Total	670 (100)

**Table 2:** Distribution of first-time blood donors and repeated blood donors

		First-time donors (*n*, %)	Repeated donors (*n*, %)	χ2	*p* value
Gender	Male	255 (85.57)	43 (14.43)	0.459	0.498
	Female	325 (87.36)	47 (12.64)		
School type	University	173 (80.09)	43 (19.91)	11.491	0.001
	Higher vocational college	407 (89.65)	47 (10.35)

**Table 3:** Comparison between the number of studied responses and total population

		Total population	The number of studied responses	χ2	*p* value
Gender	Male	3206	298	0.035	0.852
	Female	3947	372		
Donation frequency	First-time	6077	580	1.252	0.263
	Repeated	1076	90		

In study participants, we found that there was no statistically significant difference in gender between first-time donors and repeated donors (χ2 = 0.459, *p* = 0.498), but had a significant difference in school type (χ2 = 11.491, *p* = 0.001), which indicated that the proportion of blood donors in universities is higher than that in vocational colleges.

### Exploratory Factor Analysis

Exploratory factor analysis was performed on all items in the questionnaire. Kaiser–Meyer–Olkin (KMO), an indicator which is between 0 and 1, was used to compare the simple correlation coefficient and partial correlation coefficient between variables. As a result, KMO was 0.966 and the cumulative variance contribution rate was 75.489%, which means that correlation between variables is strong and the variables are suitable for factor analysis. Rotating them by the method of varimax. After rotation, all items can be obtained into 4 factors: Factor 1 has a high factor loading on 8 items, reflecting attitude; Factor 2 has a high factor loading on 4 items, reflecting blood donation external motivation; Factor 3 has a high factor loading on 6 items, reflecting advice-taking (Factor 2 and Factor 3 reflect subjective behavior norms together); Factor 4 has a high factor loading on 11 items, reflecting perceived behavioral control.

### Model Establishment

**Fig. 1:**
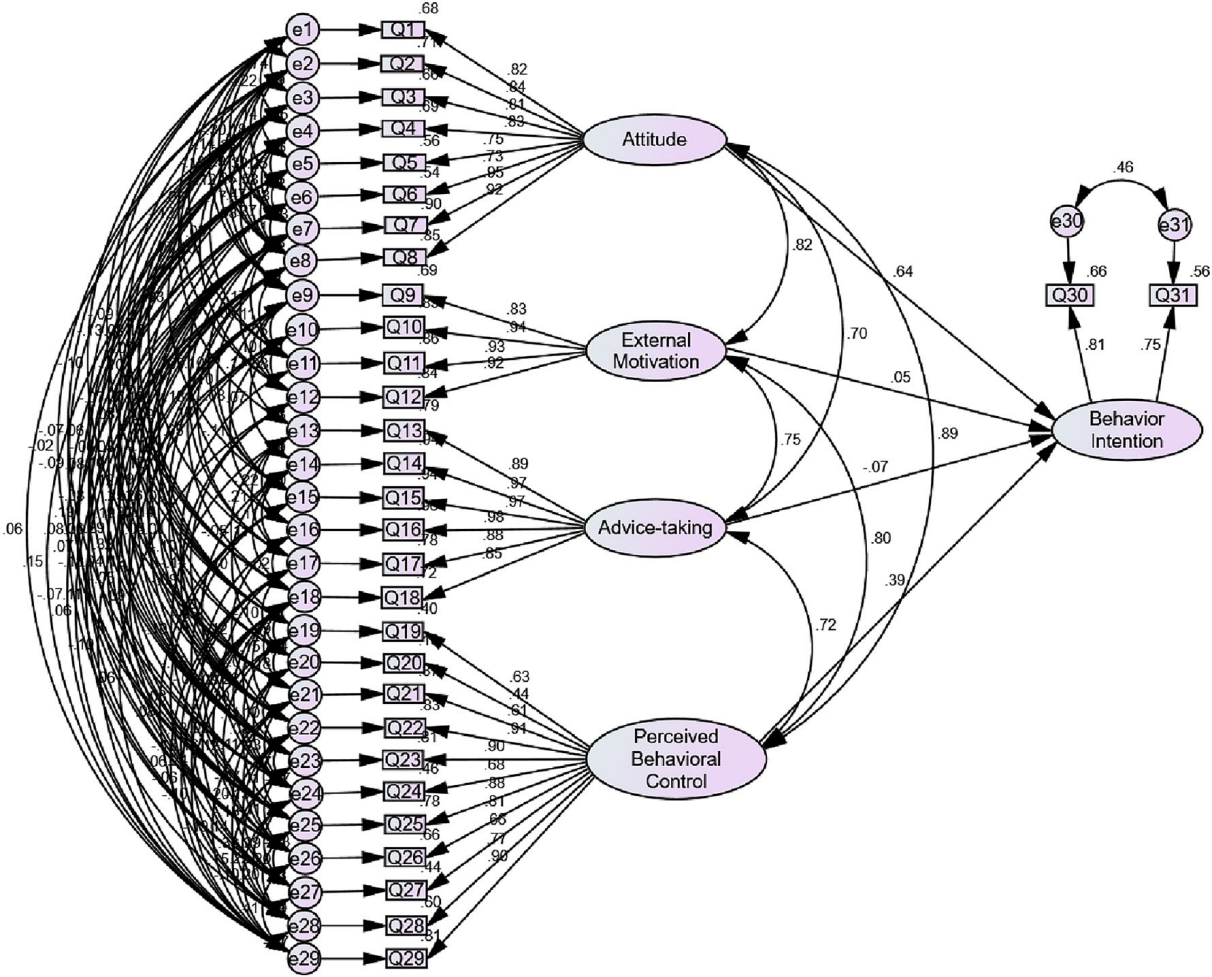
Structural equation modeling for the influencing factors of college students’ blood donation behavior intention This figure was created by Amos 21.0 software

Using Amos21. 0 software to build a preliminary model, and then performed Maximum Likelihood to evaluate it. Adjusting the path relationship between items based on modified index (MI) and evaluating the model’s adaptability. At last, using standardized methods to estimate parameters and construct the optimal model for reflecting the influencing factors of college students’ blood donation behavior intention. Figure 1 presents the structural equation modeling.

Testing the model’s adaptability by calculating CMIN/DF (χ^2^/df), root-mean-square residual (RMR), root-mean-square error of approximation (RMSEA), comparative fit index (CFI), goodness of fit index (GFI), normed fit index (NFI), and Tucker-Lewis index (TLI), the results showed that χ^2^/df = 2.956 < 3 and other indexes filled in the optimal standard range, which indicating the model with a high adaptability. (Table 4) According to the results of exploratory factor analysis and model adaptability test, it can be proved that the model is consistent with the behavioral influencing factors proposed by the theory of planned behavior.

**Table 4:** Adaptability test results of sem of influencing factors of college students’ behavior intention of blood donation

Statistics	χ^2^/df	RMR^1^	RMSEA^2^	CFI^3^	GFI^4^	NFI^5^	TLI^6^
Model adaptability test results	2.956	0.034	0.054	0.980	0.934	0.971	0.964
Optimal standard value	< 3	< 0.08	< 0.08	> 0.9	> 0.9	> 0.9	> 0.9

^1^Root-mean-square residual

^2^Root-mean-square error of approximation

^3^Comparative fit index

^4^Goodness of fit index

^5^Normed fit index

^6^Tucker-Lewis index

Calculating path coefficient for each item. (Table 5). The results showed that attitude and perceived behavior control have a greater impact on behavioral intentions, with standardized path coefficients of 0.638 and 0.392, respectively. All of the four factors are correlated and can influence each other.

**Table 5:** Load coefficient analysis result

Factor (latent variable)	Item (observed variable)	Path Coefficients
Behavior Intention	Attitude	0.638
	Perceived behavioral control	0.392
	Blood donation external motivation	0.055
	Advice-taking	− 0.069
Attitude	Blood donation external motivation	0.824
	Advice-taking	0.700
	Perceived behavioral control	0.889
Blood Donation External Motivation	Advice-taking	0.746
Perceived Behavioral Control	Advice-taking	0.721
	Blood donation external motivation	0.802
Attitude	Q7: I consider donating blood as a way of life and will donated more in the future	0.949
	Q8: I think blood can’t be synthesized, and blood donation is the only source of blood transfusion	0.920
	Q2: I think blood donation is honorable and proud	0.842
	Q4: I will promote the benefits of blood donation to others	0.831
	Q1: I think participating in blood donation is good for the society	0.825
	Q3: I think donating blood can save others’ lives	0.810
	Q6: I think blood donation shows that I am brave	0.734
	Q5: I think blood donation is a guarantee of blood storage for myself and my family	0.751
Blood Donation External Motivation	Q10: My classmates or teachers support me to donate blood	0.944
	Q11: My friends support me to donate blood	0.928
	Q12: My organization supports me to donate blood	0.916
	Q9: My family members support me to donate blood	0.833
Advice-taking	Q16: I am willing to donate blood with the recommendation from my organization	0.981
	Q15: I am willing to donate blood with the advice from my friends	0.970
	Q14: I am willing to donate blood with the advice from my classmates or teachers	0.968
	Q13: I am willing to donate blood with the advice from my family	0.888
	Q17: I am willing to donate blood with the advice from physicians	0.884
	Q18: I am willing to donate blood with the advice from public figures	0.849
Perceived Behavioral Control	Q22: I will donate blood again when the blood stock is not enough in case of an emergency	0.909
	Q23: I will donate blood again if the donation location is easy to get	0.901
	Q29: I will donate blood again if my relatives or friends need blood	0.899
	Q25: I will donate blood again because last donation experience was good	0.882
	Q26: I will donate blood again if my family can have priority to blood transfusion	0.812
	Q28: I will donate blood again if the donation procedure is easy and convenient	0.773
	Q24: I will donate blood again if I receive a reminder phone call or message	0.676
	Q27: I will donate blood again if the donation drive attracts me	0.663
	Q19: I will donate blood again when I am healthy	0.632
	Q21: I will donate blood again on special anniversary, such as birthday, wedding anniversary and so on	0.608
	Q20: I will donate blood again when I have enough time	0.439
Behavior Intention	Q30: I will donate blood again in the future	0.813
	Q31: I would suggest others to donate blood	0.746

Meanwhile, loading factor analysis results showed that: (1) To Factor 1 (Attitude), 2 items: “I consider donating blood as a way of life and will donate more in the future” and “I think blood can’t be synthesized, blood donation is the only source of blood transfusion”, had higher effects, with the path coefficients of 0.949 and 0.920, respectively. (2) To Factor 2 (Blood Donation External Motivation), 2 items: “My classmates or teachers support me to donate blood” and “My friends support me to donate blood”, had higher effects, with the path coefficients of 0.944 and 0.928, respectively. (3) To Factor 3 (Advice-taking), 2 items: “I am willing to donate blood with the recommendation from my organization” and “I am willing to donate blood with the advice from my friends”, had higher effects, with the path coefficients of 0.981 and 0.970, respectively. (4) To Factor 4 (Perceived Behavioral Control), 2 items: “I will donate blood again when the blood stock is not enough in case of an emergency” and “I will donate blood again if the donation location is easy to get”, had higher effects, with the path coefficients of 0.909 and 0.901, respectively.

## Discussion

Voluntary non-remunerated blood donation recruitment is a common puzzle over the world, many scholars in both developed and developing countries have carried out a large number of researches on recruitment and retention of blood donors [10–12]. Repeated and regular blood donors are considered as the safest source of blood because they have the lowest risk of transmitting infectious diseases. Comparing with elder people, college students are young and having good health condition, they are ideal target for regular blood donor recruitment. Therefore, studying the influencing factors of college students’ repeated blood donation behavior and improving their repeated donation rate have great significance for building a stable group of non-remunerated blood donors.

At present, many domestic and foreign scholars have conducted researches on the behavioral intention of college students and found that the factors affecting college students’ behavior include not only physiological and psychological factors, but also environmental impact on individuals [13–15]. Mills, a sociologist, pointed out that group members are inseparable from their surroundings, both mentally and physically [14, 15]. Especially in modern society, college students are more deeply involved in social activities, have stronger influence from environmental factors. Therefore, factors that affect the behavioral intention of college students’ blood donation behavior are complex and diverse. With the help of the theory of planned behavior, we can explain and predict people’s blood donation intention well by constructing structural equation modeling, better understand the internal psychological mechanism influencing college students, and provide psychological basis and empirical support for the recruitment of non-remunerated blood donors [16].

According to the questionnaire survey, attitude is the most important factor that influencing blood donation intention of college blood donors. To attitude, some items in the model, like “considering donating blood as a way of life”, “blood donation is the only source of blood transfusion”, “blood donation is honorable and proud”, had higher effects. This tells that college students have correct understanding on blood donation, they have made a connection between blood donation and healthy lifestyle, personal life value, and social justice. Therefore, we should adjust recruitment strategies in the future, not only publish blood donation knowledge by some new media favored by college students, such as social media, but also provide services based on blood donors’ needs to help them make personal health management and create more personal value.

To perceived behavioral control, some items like “I will donate blood again when the blood stock is not enough in case of an emergency”, “I will donate blood again if the donation location is easy to get”, “I will donate blood again if my relatives or friends need blood”, had higher effects on college students’ donation behavior. It shows that on the one hand, blood donors are full of sympathy. On the other hand, they are self-interested and consider their own needs when an emergency happens to them or their family members, so recruiters should adjust recruitment strategies and incentive measures based on blood donors’ needs. For example, we should guide blood donors giving blood orderly in case of a large emergency, set blood collection locations which are easy to get, follow up after donors donating blood and encourage them to become regular donors. We should also pay attention to the family members of patients who need blood transfusions, because they have a high level of acceptance on the importance of blood in that case.

The path coefficients of model showed that although blood donation external motivation and advice-taking have little influence on donation intention, they can indirectly affect it by influencing other factors. Suggestions from friends, classmates, teachers and organizations have a high effect on intentions and behaviors of college blood donors, both in external motivation and advice-taking. Thus, we can apply relationship marketing, a concept from precision marketing, to help us develop interventions for college blood donation drives [17]. If blood centers get support from college leaders and teachers, improve awareness and participation rate of blood donation among them, it will be beneficial to improve their repeated donation rate.

In this study, we used a questionnaire survey and constructed structural equation modeling for college blood donation population, to find out relevant factors that affect their donation behavior. Based on education and psychology theories, intervention strategies are formulated for different behavior intentions of college students. However, this work had some limitations that need to be taken into consideration due to under-representation of survey samples. In the follow-up study, we will expand the scope of population, conduct verification through on-site interviews and phone-call recruitment, optimize recruitment measures, improve the rate of repeated blood donation in colleges.
